# Confirmation and Fine Mapping of a Major QTL for Aflatoxin Resistance in Maize Using a Combination of Linkage and Association Mapping

**DOI:** 10.3390/toxins8090258

**Published:** 2016-09-02

**Authors:** Yu Zhang, Min Cui, Jimin Zhang, Lei Zhang, Chenliu Li, Xin Kan, Qian Sun, Dexiang Deng, Zhitong Yin

**Affiliations:** 1 Jiangsu Key Laboratory of Crop Genetics and Physiology/Co-Innovation Center for Modern Production Technology of Grain Crops, Key Laboratory of Plant Functional Genomics of the Ministry of Education, Yangzhou University, Yangzhou 225009, China; zhangyu2791@163.com (Y.Z.); yzumincui@163.com (M.C.); VIPshiyanzhanghao@163.com (L.Z.); chencai9596@163.com (C.L.); xink_abc@163.com (X.K.); m18362825831@163.com (Q.S.); yzdxdeng@126.com (D.D.); 2 Zhenjiang BGI Fisheries Science & Technology Industrial Company Limited, Zhenjiang 212000, China; m18252714993_1@163.com

**Keywords:** *Aspergillusflavus* (*A. flavus*), genome-wide association analysis (GWAS), linkage mapping, maize, molecular marker, quantitative trait locus (QTL), recombinant inbred line (RIL)

## Abstract

Maize grain contamination with aflatoxin from *Aspergillus* *flavus* (*A. flavus*) is a serious health hazard to animals and humans. To map the quantitative trait loci (QTLs) associated with resistance to *A. flavus*, we employed a powerful approach that differs from previous methods in one important way: it combines the advantages of the genome-wide association analysis (GWAS) and traditional linkage mapping analysis. Linkage mapping was performed using 228 recombinant inbred lines (RILs), and a highly significant QTL that affected aflatoxin accumulation, *qAA8*, was mapped. This QTL spanned approximately 7 centi-Morgan (cM) on chromosome 8. The confidence interval was too large for positional cloning of the causal gene. To refine this QTL, GWAS was performed with 558,629 single nucleotide polymorphisms (SNPs) in an association population comprising 437 maize inbred lines. Twenty-five significantly associated SNPs were identified, most of which co-localised with *qAA8* and explained 6.7% to 26.8% of the phenotypic variation observed. Based on the rapid linkage disequilibrium (LD) and the high density of SNPs in the association population, *qAA8* was further localised to a smaller genomic region of approximately 1500 bp. A high-resolution map of the *qAA8* region will be useful towards a marker-assisted selection (MAS) of *A. flavus* resistance and a characterisation of the causal gene.

## 1. Introduction

Maize (*Zea mays* L.) is an important worldwide crop that serves as an essential source for food, feed and fuel. *Aspergillus* *flavus* (*A. flavus*) infection poses a grave threat to maize production, resulting in ear or kernel rot and aflatoxin accumulation in the kernel [1]. The *A. flavus*-secreted aflatoxin is a well-known and serious health hazard to animals and humans that is associated with increased mortality in farm animals and an increased liver cancer incidence in humans [2,3]. Maize grain with aflatoxin levels greater than 20 ng/g is banned from interstate commerce by the U.S. Food and Drug Administration [4]. Additionally, multiple countries will not buy grain with aflatoxin levels exceeding 5 to 10 ng/g [5,6]. Thus, reducing aflatoxin contamination has become a key goal for maize breeding and production.

Numerous methods, including plant breeding and biological control, have been exploited to improve maize resistance to *A. flavus* [3]. The breeding of maize varieties for *A. flavus* resistance is currently considered an effective and environmentally safe method for controlling contamination. However, the quantitative nature and strong genotype-environment interaction of this trait limit the ability to successfully transfer resistance genes to commercial hybrids using traditional breeding methods [1,7]. The identification of *A. flavus* resistance quantitative trait loci (QTL) or genes, the discovery of closely linked markers, and the development of marker-assisted selection (MAS) methods would accelerate resistance breeding efforts.

QTL mapping is an effective genomic approach for identifying the possible causal genes that underlie a complex phenotypic trait. Multiple studies have identified numerous QTLs for *A. flavus* resistance in maize using traditional QTL linkage mapping [8,9,10,11,12,13,14,15,16,17]. While these studies provide useful information about the genetic loci for aflatoxin resistance, it is typically difficult to isolate candidate genes based on a single QTL mapping experiment. One reason for these limitations may be the traditional linkage mapping method, which has a relatively low genome resolution unless large mapping populations are used [18] there by severely limiting its use in MAS. Fortunately, this limitation can be overcome with genome-wide association analysis (GWAS), which enables increased mapping resolution from the QTL interval to the candidate gene level. The high diversity and rapid LD decay in maize enables GWAS to provide a higher mapping resolution in this species when high-density and genome-wide DNA markers are available [19]. Additionally, GWAS permits an immediate evaluation of multiple alleles in multiple genetic backgrounds. GWAS has recently been employed to detect markers that are significantly associated with *A. flavus* resistance in two maize association populations that consist of diverse germplasm collections [20,21]. However, the population structure in the germplasm collections used for the GWAS may identify false positive correlations between the polymorphic loci and phenotype, even though several models have been developed to correct the population structure [22]. The combination of GWAS and traditional linkage mapping is considered preferable for dissecting complex traits [23]. 

On the other hand, interactions with the environment may cause aflatoxin resistance QTLs or genes to become genetically unstable in different experiments. Thus, few of the previously reported aflatoxin resistance QTLs or genes were stably expressed across different environments or in different genetic backgrounds. Additionally, because gene expression depends on the genetic background, some resistance genes might have been overlooked in previous attempts to map *A. flavus* resistance, despite the diversity of the maize germplasms used in the GWAS [20,21]. For breeding purposes, the only highly valuable resistance genes are those that can be expressed in different genetic backgrounds and/or under multiple environments. Thus, a GWAS analysis using different association populations and/or a linkage mapping analysis using multiple bi-parental populations to confirm previously mapped genes and identify new *A. flavus* resistance genes is warranted.

In our previous study using a maize RIL population, we evaluated the score for kernel resistance to *A. flavus* infection (RAI) and identified eight QTLs for this trait [17]. However, the RAI score and the amount of aflatoxin (AA) reflect different aspects of *A. flavus* resistance in maize; the AA in the kernels causes more damage to maize. In this study, we measured the AA in the same RIL population and the AA and RAI score in a maize association population that comprised 437 inbred lines with tropical, subtropical and temperate backgrounds [24]. A major QTL for *A. flavus* resistance was confirmed and refined by combining the linkage analysis and GWAS approaches. Moreover, several putative genes responsible for this major QTL were identified using the maize reference genome.

## 2. Results

### 2.1. Quantitative Variation of the AA and RAI Score in Maize Populations

**Figure 1:**
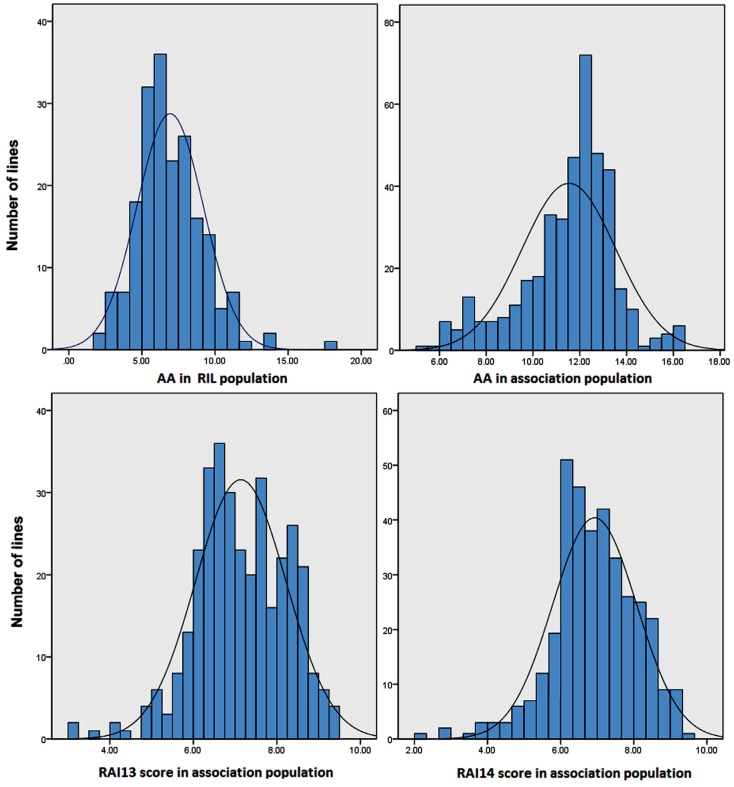
Frequency distribution of natural variations in the amount of aflatoxin (AA, ug/kg) and the score of resistance to *A. flavus* infection (RAI).

The means, standard deviations, skewness, kurtosis, ranges and broad-sense heritability values, and analyses of variance (ANOVA) for the AA and RAI score are presented in Table 1. The ANOVA indicated that the AA and RAI score were significantly affected by plant line genotype (*p* < 0.01). The AA in the two populations and the RAI score in the association population displayed a wide range. Compared with the resistant RA line, the susceptible line (M53) had a significantly higher AA, and the transgressive segregation of this trait was apparent in the RIL population. For the AA, there was a 7.4-fold difference between the lines in the RIL population and a 3.4-fold difference between the lines in the association population. For the RAI score, there was a 3.3-fold difference between the lines in the association population in 2013 and a 4.6-fold difference between the lines in the association population in 2014. The AA and RAI score showed a similar variation range in the association population compared to the RIL population. The RAI score variation range for the RIL population is described in our previous study [17]. The AA distribution in the RIL population and the AA and RAI score in the association population were relatively normal (Figure 1) and consistent with the small absolute values of skewness and kurtosis (less than 1.0) for the two traits (Table 1). The heritability (*h*^2^) estimates for the AA and RAI score ranged from 78% to 85% (Table 1). Overall, the maize plants clearly exhibited considerable natural variations in the AA and RAI score and displayed a high genetic diversity.

### 2.2. Phenotypic Correlations between the AA and RAI Score in Maize Populations

A correlation analysis was performed for the AA and RAI score (Table 2). In the RIL population, there was a significant positive correlation between the AA and RAI score. In the association population, the AA showed a significant positive correlation with the RAI score in both 2013 and 2014, and the 2013 RAI score was significantly correlated with that of 2014.

### 2.3. QTL Mapping for AA Using Linkage Analysis

**Figure 2:**
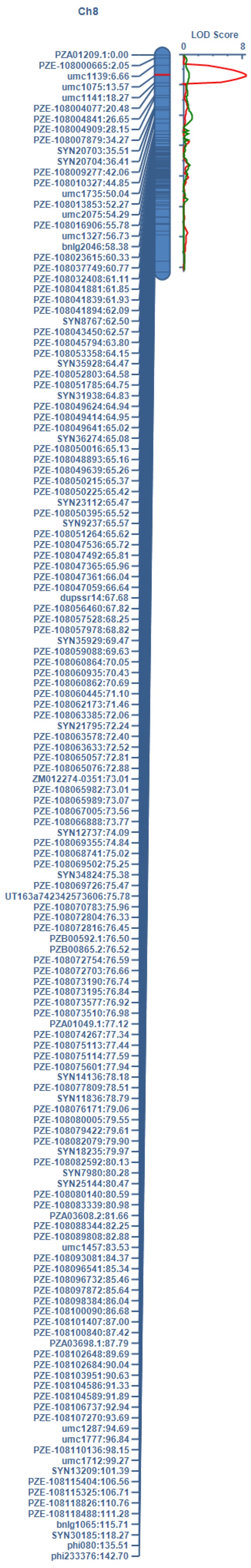
Quantitative trait loci (QTL) for the amount of aflatoxin (AA, μg/kg) on maize chromosome 8. The designation on the left is the genetic distance (centi-Morgan, cM) and marker name. The right shows the likelihood odds (LOD) scores of the QTL on the chromosome.

The QTL analysis revealed one significant additive QTL for the AA—qAA8—on chromosome 8 (Table 3, Figure 2). This QTL explained 18.23% of the phenotypic variation. Moreover, this QTL was previously implicated in controlling the RAI score in the same RIL population [17]. The additive effect values indicated that the resistant parent RA alleles decreased both the AA and RAI score at this locus. Thus, this QTL may be a target region for identifying genes associated with an improved *A. flavus* resistance. 

**Figure 3:**
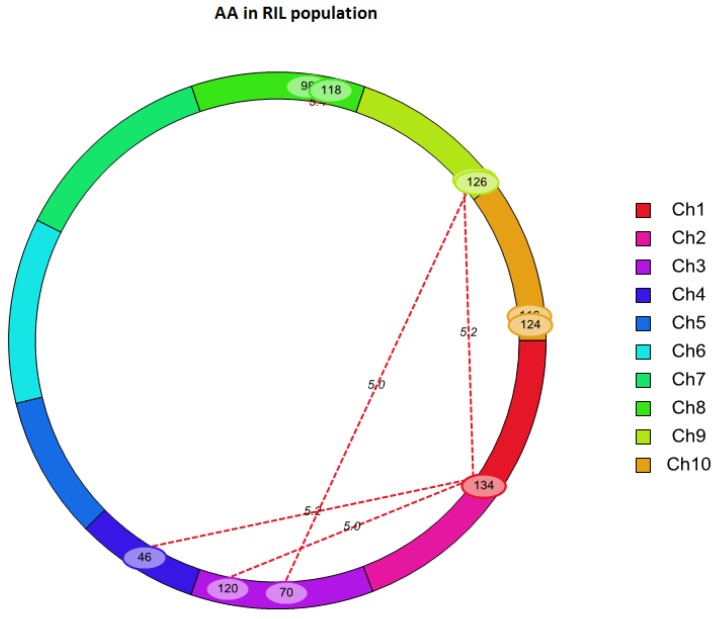
Epistatic effects on the amount of aflatoxin (AA). The lines denote epistatic associations between QTLs.

To examine epistatic interactions, six pairs of interacting QTLs were mapped to chromosomes 1, 3, 8 and 10 (Table 4, Figure 3). Two pairs of epistatic QTL interactions on chromosomes 8 and 10 reduced the AA by 1.51 and 1.75 μg per kilogram of maize kernel, respectively, whereas the remaining pairs increased the AA level. The phenotypic variation explained by these interactions ranged from 14% to 23%. The additive QTL on chromosome 8, qAA8, did not show a significant epistatic interaction.

### 2.4. Association Mapping for the AA and RAI Score

GWAS was performed with 558,629 single nucleotide polymorphisms (SNPs), and the phenotypic AA and RAI score values were generated in an association population comprising 437 inbred maize lines to identify the loci associated with A. flavus resistance. To account for spurious associations that might arise from the historical relationships and selection patterns of the inbred lines in the association population, three statistical models were evaluated. A visual observation of the quantile-quantile (Q-Q) plots illustrates the accuracy of the model used to analyse the data. As shown in the Q-Q plots (Figure S1), the GLM model (considering Q) was suitable for reducing the effect of population structure on the maize AA and RAI score. The P values from this model are close to the expected values, indicating that this model is suitable for the association analysis. Therefore, we conducted the GWAS for the maize AA and RAI score with the GLM + Q model to correct for population structure.

**Figure 4:**
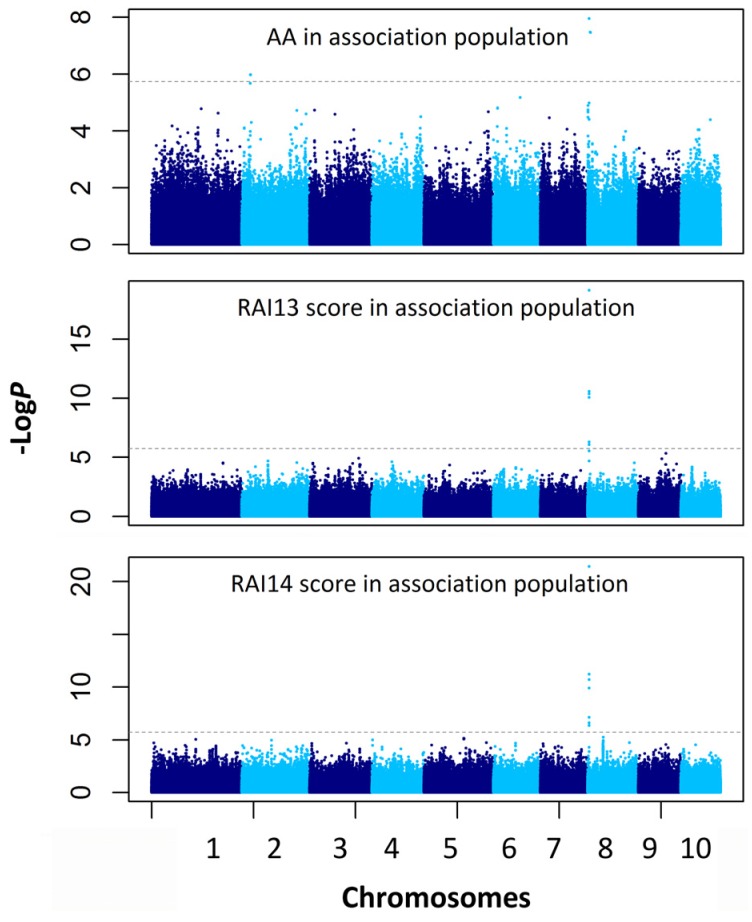
Genome-wide association mapping of the amount of aflatoxin (AA, μg/kg) and the score of resistance to *A. flavus* infection (RAI) based on SNPs. The dashed line indicates a significant association signal (−log*P* ≥ 5.74).

Three SNPs for the AA and 22 SNPs for the RAI score were identified as having significant marker-trait associations at the Bonferroni-adjusted significance threshold (−log*P* ≥ 5.74) (Table 5, Figure 4). The three significant SNPs associated with the AA were located on chromosomes 2 and 8 and explained 6.7%–10.4% of the phenotypic variation. Of the 22 significant marker-trait associations for the RAI score, 10 and 12 were detected in 2013 and 2014, respectively. The significant SNPs for the RAI score were located on chromosomes 5, 8, and 9 and explained 6.4%–26.6% of the phenotypic variation. The highly significant SNP cluster on chromosome 8 (which included four SNPs, *p* = 3.7 × 10^−22^) was repeatedly detected for the RAI score in 2013 and 2014. As with the RIL population, this SNP cluster was co-localised with the significant SNP markers for the AA in the association population. Importantly, this significant SNP cluster fell within the QTL region detected for the AA by linkage analysis (Table 3, Figure 2).

To reduce the major QTL qAA8 region detected in the RIL population, we focused on the QTL region in the association population. Altogether, there were nine SNPs located in the AA and RAI score QTL region in the association population; this QTL was co-localised with qAA8 on chromosome 8. Linkage disequilibrium (LD) was estimated from the *r*^2^ correlation between each marker in this region and the closest neighbouring SNPs (approximately 1 Mb in the upstream and downstream directions) in the maize genome (Figure S2). *r*^2^ values less than 0.2 were considered unlinked. Three LD blocks that contained the significant SNPs and extended to approximately 1500 bp were observed. These blocks contained a candidate gene (GRMZM2G074857) that encoded a BolA-like protein.

## 3. Discussion

Maize grain contamination by aflatoxin has major economic and health implications that include increases in the disease burden and mortality rate throughout the developing world and income losses across the developed world [3]. In the last decade, efforts have been made to dissect the genetic basis of maize aflatoxin resistance, and multiple aflatoxin resistance-associated QTLs have been identified across the 10 maize chromosomes [16,20]. However, most of these QTLs have exclusively been detected in one environment where the majority of the QTLs individually account for less than 5% of the phenotypic variation. This finding has led to difficulties in selecting suitable candidate genes for aflatoxin resistance and in applying this knowledge towards breeding [3]. The main reasons behind these limitations may be inappropriate phenotyping, environmental effects, and/or the large genomic region of the identified QTLs.

### 3.1. Direct Inoculation and Cultivation of A. flavus Fungi on Maize Kernels under Controlled Conditions Is an Accurate Method for Phenotypic Identification

A precise phenotypic evaluation is a prerequisite for QTL mapping. In this study, *A. flavus* fungi were inoculated and grown on maize kernels under controlled conditions as described in our previous study [17]. This phenotyping method has been used in our laboratory for many years [25,26,27]. We adopted the method from Professor Manjit S. Kang of the Louisiana State University Agronomy Department in 1991 and have continuously improved this method over the last two decades. The method has proved to be reliable and applicable [17,25,26,27]. Controlled conditions can minimize the phenotyping errors caused by the highly variable environmental factors in field conditions to provide a precise phenotypic evaluation. As a result, we observed high variation levels in the AA and RAI score in both the RIL and association population (Table 1), and the heritability values for the two traits were higher than those from most studies previously conducted in field conditions (0.20–0.60) [8,9,10,11,12,13,14,15]. The higher heritability values in this study (0.78–0.85, Table 1) were similar to those calculated in two recent studies that used testcrossed maize hybrids to improve the evaluation of *A. flavus* resistance [20,21]. The high variation level within the maize population and the high heritability of the AA and RAI score indicated that the data collected for these two traits in this study were suitable for a linkage and association mapping analysis.

Established methods using field inoculations have been widely used to investigate maize resistance to *A. flavus* [3,8,9,10,15,20]. Although we used controlled conditions for phenotyping, the indicator of resistance examined in this study should be somewhat reflective of the indicators employed in the field inoculation experiments of other studies. The common factors underlying *A. flavus* resistance in controlled conditions and field conditions might rely on maize kernel characteristics. Previous studies have suggested that multiple kernel characteristics, including kernel proteins, kernel wax, kernel surface cutin layers, and other unknown compounds in the kernels, may have important effects on an *A. flavus* infection and its subsequent aflatoxin production [9,28].

### 3.2. Confirmation and Fine Mapping of a Major QTL for Maize A. flavus Resistance Breeding

*A. flavus* causes ear rot and a significant aflatoxin accumulation in maize, particularly in southern growing regions [29,30]. In our recent study, which utilized the same RIL population as our current study, we identified eight QTLs for the RAI score [17]. Because the RAI score directly reflects the fungal colonisation of kernels by *A. flavus*, we suggest that these eight QTLs may be associated with ear rot resistance. Considering that AA is more related to economic losses in maize and the health of humans and animals, we also mapped the QTL for this trait in the same RIL population. We observed that the QTL for the AA on chromosome 8 (*qAA8*) in this study (Figure 2, Table 3) also controlled the RAI score in our previous study. Moreover, the desirable alleles at this locus originated from the same parent line, RA. This QTL region was also significantly associated with both the AA and RAI score in the association population (Figure 4, Table 5). These results suggested that the *qAA8* region had possible pleiotropic effects on both resistance traits. Furthermore, this QTL was also determined to control the AA in an F2:3 mapping population derived from Mp313 × Va35 [15] and an RIL population derived from B73 × CML322 [16]. Thus, this QTL can be considered as a major QTL for *A. flavus* resistance, which should be useful for breeding efforts to reduce *A. flavus*-mediated ear rot and aflatoxin contamination in maize.

The markers that are closely linked to the important QTLs can be used for MAS breeding. However, the success of MAS breeding depends on the gene mapping resolution and germplasm diversity. In this study, the major QTL for *A. flavus* resistance, *qAA8*, was mapped at a low resolution (approximately 7 centi-Morgan, cM) using linkage analysis (Figure 2 and Table 3). GWAS was performed to refine the position of this QTL. Based on the high density of SNPs and rapid LD decay around the peak-associated SNP marker (chr8.S_3662578, *p* = 10^−22^) in the association population, *qAA8* was delimited to a region of approximately 1500 bp on chromosome 8 (Figure S2 and Table 5). This interval was narrower than the intervals identified in previous studies [15,17], in which the average sizes of related QTL intervals exceeded 7 cM according to the linkage maps. Additionally, the GWAS enabled a search for favourable alleles in locally adapted germplasms with diverse genetic backgrounds. In this study, a base transition (A/G) in the SNP marker chr8.S_3662578 was highly associated with maize *A. flavus* resistance and led to a 36% to 42% increase in the RAI score and a 30% increase in the AA in different lines (Figure S3). This polymorphism corresponded to a variance in the trait. Thus, it can be considered a candidate site for functional molecular markers. Although this marker would be useful in MAS, further confirmation is necessary because the marker alleles are correlated with, but not entirely predictive of, the gene alleles.

### 3.3. Candidate Genes Predicted for Maize A. flavus Resistance

Proteomic studies have shown that multiple stress-related proteins may play potentially important roles in maize kernel resistance to *A. flavus* infections or aflatoxin production [28]. Based on our study’s high-density GWAS and LD analyses, the genomic region on chromosome 8 that contained the causal gene in *qAA8* had a physical distance of approximately 1500 bp (Table 5, Figure S2). To identify candidate genes that affect *A. flavus* resistance, the annotated genes around this genomic region were investigated using an annotation of the B73 maize inbred line reference genome [31]. A comprehensive analysis of the nearby region (approximately 1 Mb in the upstream and downstream directions) and its associated hot spots predicted five candidate iron-sulfur protein-encoding genes, including a BolA-like protein (*GRMZM2G074857*), a multinomial cystatin putative protein (*GRMZM2G013461*), and three hypothetical proteins (*GRMZM2G136158*, *GRMZM2G467059* and *GRMZM2G139952*). The previously characterised roles of iron-sulfur proteins in plant stress responses [32] highlight these genes as suitable candidates for *A. flavus* resistance. 

However, this candidate gene investigation might not be completely accurate because only those SNPs that matched the B73 reference genome could be used in this analysis. The resistance genes present in maize lines other than B73, which was one of the most susceptible inbred lines in the association population, might have been missed. Furthermore, many factors involved in the kernel colour, antioxidant profile, lipid profile, protein profile, pericarp, and endosperm have been suggested to influence fungal infections and the subsequent fumonisin accumulation in maize kernels [33]. Similarly, these factors could also play roles in determining the resistance of maize kernels to *A. flavus*. Therefore, further studies focusing on a map-based cloning and verification of the causal genes (e.g., using CRISPR/Cas9 gene editing technology) within *qAA8* are warranted. 

## 4. Conclusions 

In summary, we confirmed one major QTL that was responsible for maize *A. flavus* resistance on chromosome 8; this QTL was narrowed to an interval of approximately 1500 bp using a combined linkage and association mapping approach. Such fine-mapping of QTLs into smaller chromosomal fragments could increase the value of QTLs in MAS for maize *A. flavus* resistance by reducing the potential for linkage drag. Additionally, the results of this study provide important information for a map-based cloning of the genes responsible for maize *A. flavus* resistance and will be a useful reference for further candidate gene research into complex resistance traits.

## 5. Materials and Methods 

### 5.1. Plant Materials and Plant Growth Conditions

The RIL population used to map QTLs was derived from a cross between the maize RA and M53 inbred lines via single seed descent. This population comprised 228 F8:9 lines and has been used to map QTLs for the RAI score [17]. The two parental lines, RA and M53, were selected based on our previous resistance identification results [26,27]. RA was developed from a cross between two Chinese elite inbred lines, Ye478 and Dan340. M53 was developed from a landrace from the Yunnan Province of China. The difference between these two lines upon infection with *A. flavus* has consistently been replicated in trials over multiple years [25]. The previously constructed linkage map for this RIL population covered 1367 cM of the maize genome and was converted into 10 linkage groups consisting of 916 molecular markers [17]. The average distance between markers was 1.50 cM.

For the GWAS, 437 maize lines that exhibited normal growth and maturation in Yangzhou were chosen from a previously assembled maize association population [24]. This chosen collection included 52 lines from the Germplasm Enhancement of Maize (GEM) project, 195 lines from the CIMMYT maize breeding programme and 190 lines from China. All 437 lines were genotyped for SNP markers using high-throughput genotyping platforms, and approximately 558,629 polymorphisms with minor allele frequencies (MAF) ≥ 0.05 were available for GWAS [34].

All lines were planted in the experimental farm of the Agricultural College of Yangzhou University. The RILs and parental lines were planted during the 2012 growing season. This growing season was designated as environment E3 in our previous study [17]. One replicate of each line was planted during this growing season. The plants with different genotypes were grown in single, 5 m rows that were spaced 0.67 m apart and were planted at 45,000 plants/ha. Each line was self-pollinated to produce enough kernels for the RAI score [17] and AA measurements. The association population of 437 lines was planted during two growing seasons (2013 and 2014). In each growing season, all lines were planted with two replicates in a randomized complete-block design. Each line was planted in one row per plot; the length of each row was 2.5 m, the spacing between plants in each row was 0.25 m, and the spacing between rows was 0.55 m. The plants were self-pollinated to produce enough kernels for the RAI score and AA measurements in 2013 and the RAI score measurements in 2014. The 2013 and 2014 RAI scores were designated as RAI13 score and RAI14 score, respectively. 

### 5.2. Phenotypic Evaluation

Kernels from the RIL population and its parents RA and M53 were investigated for AA. Kernels from the association population were investigated for both RAI score and AA. A randomized complete-block design with three replicates was used for trait investigation. Kernels from each genotype were divided into three groups, constituting three biological replicates. The inoculation and cultivation of *A. flavus*, and survey of RAI score were conducted according to the procedure described in our previous study [17]. This procedure was similar to that employed by Windham and Williams (1998) [35], with the main difference being that we conducted the inoculation and cultivation of the *A. flavus* fungi on harvested mature kernels under controlled conditions, while Windham and Williams performed field inoculations on maize ears. Briefly, kernels were inoculated with a conidial suspension (2 × 10^6^ conidia/mL) of *A. flavus* for 1 min, and then placed in Petri dishes (Sinopharm, Shanghai, China) with Czapek agar medium [36] for a 7-day cultivation of the *A. flavus* fungi in a chamber at 30 °C and 95% relative humidity. After removal of the surface *A. flavus*, the kernels were placed once again in new Petri dishes with Czapek agar medium. This step was to cultivate the subepidermal fungi developed in the first step of cultivation. On the 9th day of the subepidermal fungi cultivation, photos of all the treated kernels were taken and used to survey for the RAI score. The RAI score was recorded as level 0 when *A. flavus* conidia were not observed on the of kernel surface. Levels 1–10 indicated the percentage of the kernel surface that was covered by *A. flavus* conidia; the levels increased in 10% increments up to 100% for level 10. 

After determining the RAI score, the treated kernels were dried and ground for the AA measurement. In this study, the AA represented the amount of aflatoxin per kilogram of maize kernels, and the unit used for this trait was μg/kg. The AA was measured using Aflatoxin B1 ELISA kits (BestBio, Shanghai, China) according to the manufacturer’s protocol. Briefly, 2 g of the kernel powder sample were added into 4 mL of a methanol solution (70% (*v*/*v*)) to extract the aflatoxin. For each sample, the 5-μL aflatoxin extract was diluted 1:2 in the sample diluent and used for the AA measurement. To calculate the aflatoxin content in the 5-μL aflatoxin extract, standard curves were generated with aflatoxin standards. The active range of each standard curve was 0.1–8.1 ng/mL. The measurement signals for all maize lines examined in this study fell within the active range when this procedure was used.

### 5.3. Statistical Analysis and QTL Mapping

Descriptive statistical analyses, frequency distribution analyses and correlation analyses of the phenotypic data were calculated using the SPSS Statistics 17.0 statistical software package for Windows (SPSS Inc., Chicago, IL, USA). A univariate ANOVA (α = 0.05) test was used for the comparison between replicates and between genotypes. Broad-sense heritability was calculated as described by our previous study [17].

The additive and epistatic QTLs underlying the AA in the RIL population were identified using the QTL IciMapping programme v4.0 (Chinese Academy of Agriculture Sciences, Beijing, China, 2014) with single environment phenotypic values [37]. Briefly, the inclusive composite interval mapping (ICIM) method was used in the software for the additive QTLs, and the *p* values for entering variables (PIN) and removing variables (POUT) were set at 0.01 and 0.02, respectively. The scanning step was 2 cM. The ICIM-EPI method was used to detect epistatic QTLs, the PIN and POUT were set at 0.0001 and 0.0002, respectively, and the scanning step was 5 cM. The likelihood odds (LOD) thresholds for each QTL index were determined by 1000 permutation tests at the 95% confidence level. The proportion of the observed phenotypic variance explained by each additive or epistatic QTL and the corresponding additive effects were also estimated. 

The GWAS was conducted using the TASSEL software 5.0 (Cornell University, Ithaca, NY, USA, 2016) [38]. To account for the effects of the population structure on the mapping panel and genetic relatedness among panel members, the population structure (Q) and the kinship matrix (K) were calculated as previously described [24]. In the GWAS, the following three statistical models were evaluated: (1) the GLM model without the Q and K; (2) the GLM model with the Q, where the Q matrix was included as a cofactor in the regression model to correct for the population structure; and (3) the MLM model with the Q and K, which regarded population structure and kinship as cofactors. According to the quantile-quantile (Q-Q) plot from the TASSEL 5.0 output, the Q methods were appropriate for this study. Markers were identified as significantly associated with traits via comparisons using the Bonferroni threshold (*p* ≤ 1/558629 = 1.8 × 10^−6^, −log*P* ≥ 5.74).

### 5.4. Gene Prediction

Gene prediction in the target genomic region was conducted using maize genome information and bioinformatics. The related candidate genes were identified after submitting the predicted genes to a BLASTP query of the UniRef database [39] and after the synteny comparisons between the maize and other monocotyledons.

## Figures and Tables

**Table 1:** Descriptive statistics, ANOVA and broad-sense heritability for the amount of aflatoxin (AA, μg/kg) and the score of resistance to *A. flavus* infection (RAI).

Plant Material	Traits ^a^	Mean ± SD ^b^	Range ^c^	Skew	Kurt	Replicate ^d^	Genotype ^e^	*h^2^* (%) ^f^
RIL male parent M53	AA	10.70 ± 1.10	9.63–11.84					
RIL female parent RA	AA	9.07 ± 1.02	8.21–10.21					
RIL population	AA	6.93 ± 3.54	2.46–18.23	0.98	0.81	**	**	79.4%
Association population	AA	11.55 ± 2.10	4.72–16.20	−0.68	0.52	ns	**	78.2%
	RAI13 score	7.20 ± 1.23	2.92–9.67	−0.33	−0.07	**	**	83.5%
	RAI14 score	6.99 ± 1.30	2.08–9.67	−0.46	0.46	**	**	85.1%

^a^ RAI13 score and RAI14 score indicate the RAI scores measured in 2013 and 2014, respectively; ^b^ Denotes mean ± standard deviation; ^c^ Denotes the variation range from minimum to maximum; ^d,e^ Denotes the analysis of difference among genotypes and among replicates for AA or RAI score by ANOVA; ** Significant at *p* < 0.01; ns denotes a non-significant difference; ^f^ Broad-sense heritability.

**Table 2:** Phenotypic correlations between the amount of aflatoxin (AA, μg/kg) and the score of resistance to *A. flavus* infection (RAI) based on the means of the traits in the RIL and association populations.

Traits	AA in RIL Population	AA in Association Population	RAI13 Score in Association Population
RAI score in RIL population	0.33 **		
RAI13 score in association population		0.40 **	
RAI14 score in association population		0.34 **	0.72 **

The RAI score of the RIL population was determined in our previous study [17]. The RAI13 and RAI14 scores indicate the scores measured in 2013 and 2014, respectively. ** Significant at *p* < 0.01.

**Table 3:** QTL analysis for the amount of aflatoxin (AA, μg/kg) in the RIL population.

QTL	Chr.	Marker Interval	Position	LOD	ADD	*PVE* (%)
*qAA8*	8	umc1139–umc1075	42.31	8.42	0.99	18.23

ADD: additive effect; LOD: the likelihood odds; *PVE*: the percentage of phenotypic variation explained.

**Table 4:** Epistatic loci for the amount of aflatoxin (AA, μg/kg) in the RIL population.

Chr.	Left Marker	Right Marker	Chr.	Left Marker	Right Marker	LOD	*PVE* (%)	Add × Add
1	SYN7055	PZE-101199598	3	PZE-103164358	PZE-103175779	5.02	19.38	1.55
1	SYN7055	PZE-101199598	4	SYN32516	PZE-104016174	5.21	17.04	1.88
1	SYN7055	PZE-101199598	9	PZE-109104633	umc1982	5.17	17.15	1.75
3	PZE-103112971	umc1399	9	umc1982	umc1657	5.05	16.63	1.83
8	umc1777	PZE-108110136	8	bnlg1065	SYN30185	5.42	14.05	-1.51
10	SYN19288	PZE-110111130	10	PZE-110111130	PZE-110110920	5.01	22.6	-1.75

Add × Add indicates an epistatic effect for the QTLs; *PVE* represents the contribution ratio of the QTL. All estimated values were significant at a probability level of 0.005.

**Table 5:** The SNP markers associated with the amount of aflatoxin (AA, μg/kg) and the score of resistance to *A. flavus* infection (RAI) in the association population.

Traits	Chr.	Marker Position (Mb)	*P* ^a^	−log*P*	*R*^2^ ^b^
AA	8	chr8.S_3662578	1.1 × 10^−8^	7.96	0.104
AA	8	chr8.S_3353245	1.3 × 10^−8^	7.88	0.067
AA	2	chr2.S_9361865	2.1 × 10^−7^	6.68	0.079
RAI13 score	8	chr8.S_3662578	7.4 × 10^−20^	19.13	0.266
RAI13 score	8	chr8.S_3662694	2.7 × 10^−11^	10.57	0.148
RAI13 score	8	chr8.S_3662804	2.7 × 10^−11^	10.57	0.148
RAI13 score	8	chr8.S_3662702	4.3 × 10^−11^	10.37	0.154
RAI13 score	8	SYNGENTA16977	8.5 × 10^−11^	10.07	0.145
RAI13 score	8	chr8.S_3662186	4.9 × 10^−7^	6.31	0.091
RAI13 score	8	chr8.S_3662564	8.3 × 10^−7^	6.08	0.089
RAI13 score	8	chr8.S_3662567	8.3 × 10^−7^	6.08	0.089
RAI13 score	9	chr9.S_136479765	1.6 × 10^-6^	5.78	0.074
RAI13 score	9	chr9.S_136479840	1.6 × 10^−6^	5.78	0.074
RAI14 score	8	chr8.S_3662578	3.7 × 10^−22^	21.43	0.268
RAI14 score	8	chr8.S_3662694	5.9 × 10^−12^	11.23	0.144
RAI14 score	8	chr8.S_3662804	5.9 × 10^−12^	11.23	0.144
RAI14 score	8	chr8.S_3662702	2.0 × 10^−11^	10.70	0.144
RAI14 score	8	SYNGENTA16977	1.2 × 10^−10^	9.91	0.130
RAI14 score	8	chr8.S_3662564	7.4 × 10^−8^	7.13	0.096
RAI14 score	8	chr8.S_3662567	7.4 × 10^−8^	7.13	0.096
RAI14 score	8	chr8.S_3662186	2.4 × 10^−7^	6.61	0.087
RAI14 score	8	chr8.S_3662642	4.4 × 10^−7^	6.36	0.082
RAI14 score	5	chr5.S_159454183	7.2×10^−6^	5.92	0.065
RAI14 score	5	chr5.S_159758011	7.4 × 10^−6^	5.86	0.065
RAI14 score	5	chr5.S_159454148	8.7 × 10^−6^	5.78	0.064

^a^ *p* value from ANOVA analysis of the mean AA and RAI score values from three replicates; ^b^ *R^2^* value showing the percentage of phenotypic variation explained by ANOVA.

## Supplementary Materials

The following are available online at www.mdpi.com/2072-6651/8/9/258/s1, Figure S1: Quantile-quantile (Q-Q) plots of estimated −log10 (*P*). Q-Q plots for the marker-trait association analysis for the AA and RAI score were generated using the GLM + Q method. The black line is the expected line under a null distribution. The observed *p* values for the AA and RAI score are represented by the indicated colours, Figure S2: Linkage disequilibrium (LD) analysis using the *r*^2^ correlation between each marker within the association hot spots. Solid black lines represent the LD blocks, Figure S3: The association between chr8.S_3662578 marker allele polymorphisms and the AA and RAI score. Box plots for the AA (first two columns), RAI13 score (middle two columns) and RAI14 score (last two columns) in A-type and G-type maize inbred lines. The AA and RAI score of the G-type accessions were significantly higher than those of the A-type accessions (*t*-test: *p* = 7.73 × 10^−10^, 1.65 × 10^−21^, and 2.97 × 10^−25^ for AA, RAI13 score and RAI14 score, respectively).
