# Supplementary material for: Confirmation and Fine Mapping of a Major QTL for Aflatoxin Resistance in Maize Using a Combination of Linkage and Association Mapping
Source: Toxins (Basel). 2016 Sep 2;8(9):258. doi: 10.3390/toxins8090258 (PMC5037484; doi:10.3390/toxins8090258)
Supplement: Supplementary file 1 [file toxins-08-00258-s001.pdf]

Supplementary Materials: Confirmation and Fine Mapping of a Major QTL for Aflatoxin Resistance in Maize Using a Combination of Linkage and Association Mapping

Yu Zhang, Min Cui, Jimin Zhang, Lei Zhang, Chenliu Li, Xin Kan, Qian Sun, Dexiang Deng and Zhitong Yin

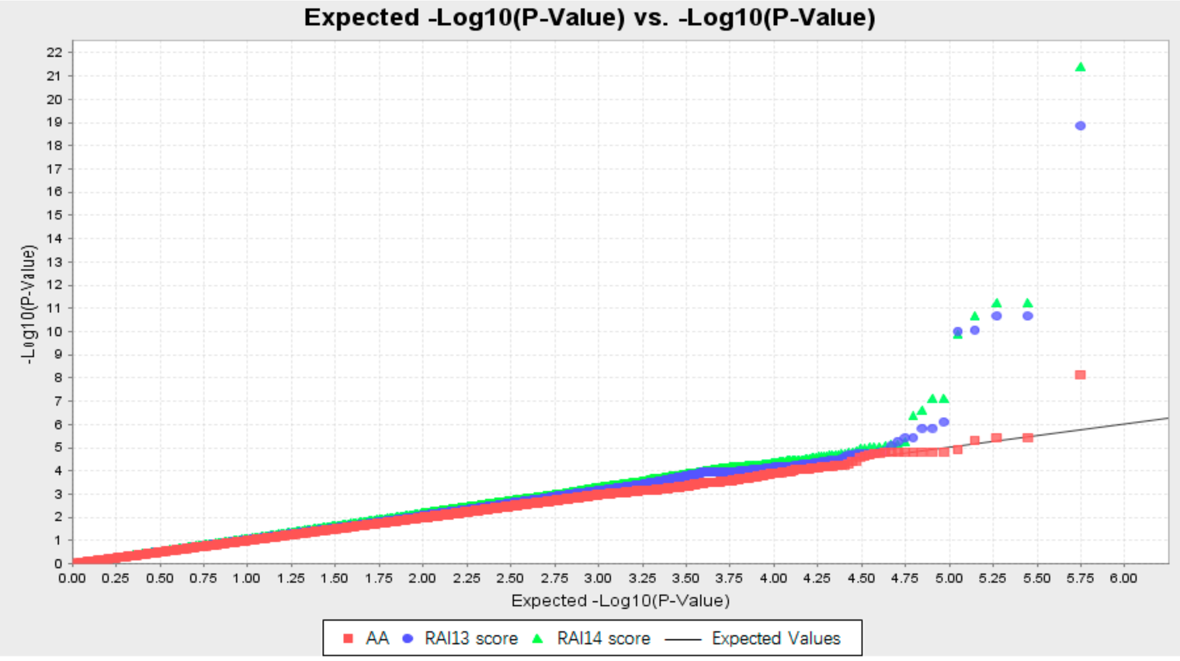

Figure S1. Quantile-quantile (Q-Q) plots of estimated −log10 (*P*). Q-Q plots for the marker-trait association analysis for the AA and RAI score were generated using the GLM + Q method. The black line is the expected line under a null distribution. The observed *P* values for the AA and RAI score are represented by the indicated colours.

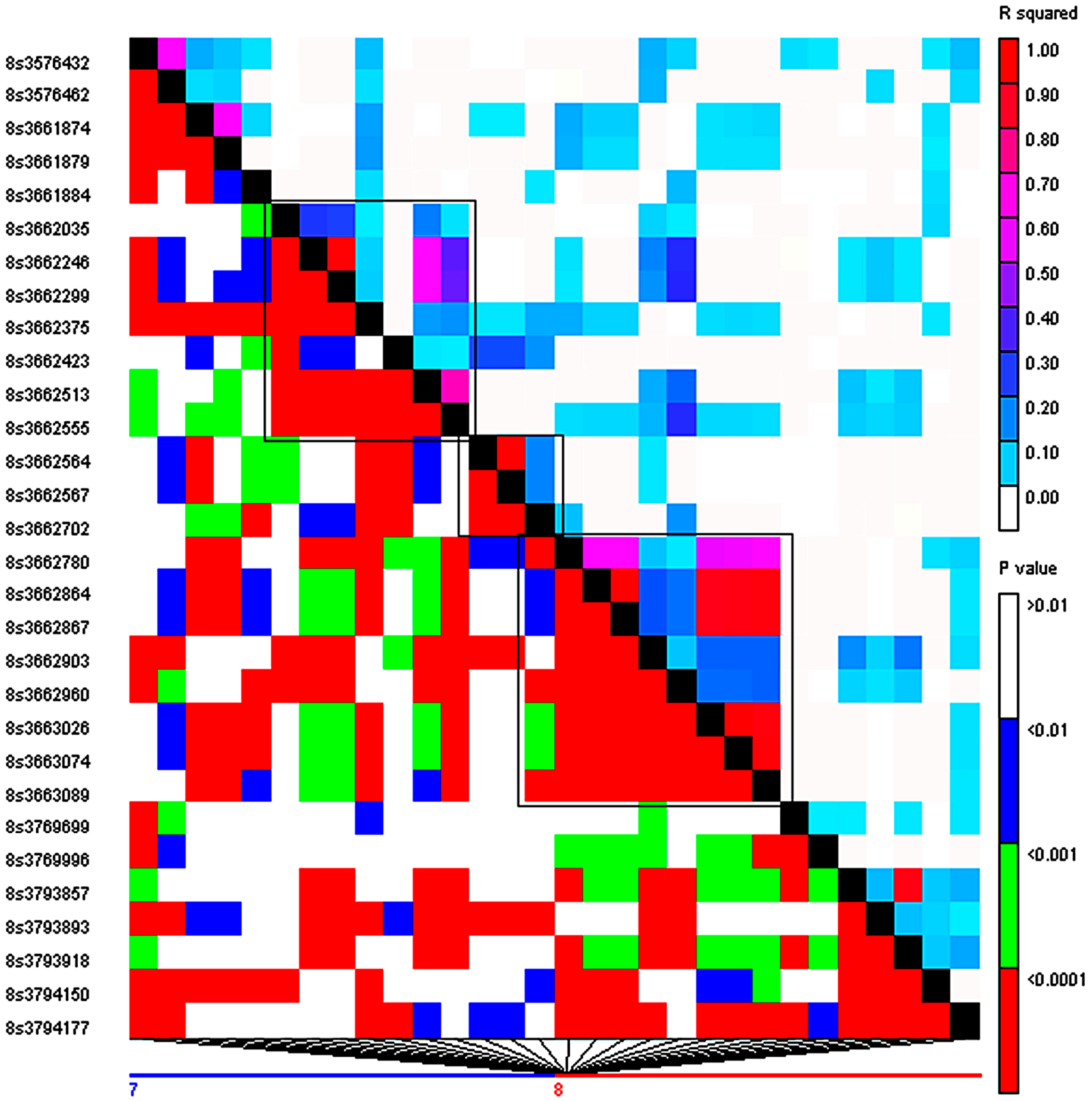

Figure S2. Linkage disequilibrium (LD) analysis using the r^2 correlation between each marker within the association hot spots. Solid black lines represent the LD blocks.

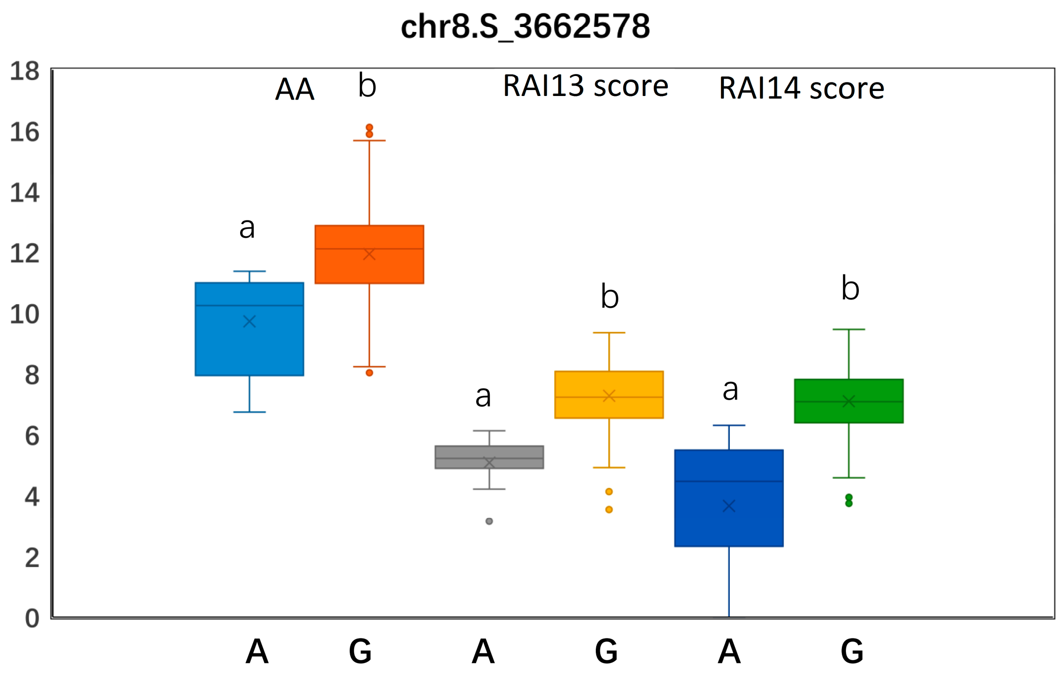

Figure S3. The association between chr8.S_3662578 marker allele polymorphisms and the AA and RAI score. Box plots for the AA (first two columns), RAI13 score (middle two columns) and RAI14 score (last two columns) in A-type and G-type maize inbred lines. The AA and RAI score of the G-type accessions were significantly higher than those of the A-type accessions (*t*-test: $p = 7.73 \times 10^{-10}$, 1.65×10^{-21}, and 2.97×10^{-25} for AA, RAI13 score and RAI14 score, respectively).
